# Copy number alteration of the interferon gene cluster in cancer: Individual patient data meta-analysis prospects to personalized immunotherapy

**DOI:** 10.1016/j.neo.2021.08.004

**Published:** 2021-09-20

**Authors:** Ali Razaghi, Nele Brusselaers, Mikael Björnstedt, Mickael Durand-Dubief

**Affiliations:** a Division of Pathology, Department of Laboratory Medicine, Karolinska Institute, Karolinska University-Hospital, Stockholm, Sweden; b Centre for Translational Microbiome Research (CTMR), Department of Microbiology, Tumor and Cell Biology, Karolinska Institute, Karolinska Hospital, Stockholm, Sweden; c Global Health Institute, Antwerp University, Belgium; d Department of Head and Skin, Ghent University, Belgium; e Department of Biosciences and Nutrition, Neo, Karolinska Institute, Huddinge, Sweden

**Keywords:** Interferon, Copy number alteration, Cancer, Mortality, Survival, CAN, copy number alteration, IFN, interferons

## Abstract

Interferon (IFN) therapy has been the standard of care for a variety of cancers for decades due to the pleiotropic actions of IFNs against malignancies. However, little is known about the role of copy number alteration (CNA) of the IFN gene cluster, located at the 9p21.3, in cancer. This large individual patient data meta-analysis using 9937 patients obtained from cBioportal indicates that CNA of the IFN gene cluster is prevalent among 24 cancer types. Two statistical approaches showed that notably deletion of this cluster is significantly associated with increased mortality in many cancer types particularly uterus (OR = 2.71), kidney (OR = 2.26), and brain (OR = 2.08) cancers. The Cancer Genome Atlas PanCancer analysis also showed that CNA of the IFN gene cluster is significantly associated with decreased overall survival. For instance, the overall survival of patients with brain glioma reduced from 93m (diploidy) to 24m (with the CNA of the IFN gene). In conclusion, the CNA of the IFN gene cluster is associated with increased mortality and decreased overall survival in cancer. Thus, in the prospect of immunotherapy, CNA of IFN gene may be a useful biomarker to predict the prognosis of patients and also as a potential companion diagnostic test to prescribe IFN α/β therapy.

## Introduction

The type-I interferons (IFN) are cytokines which play essential roles in inflammation, immunoregulation, tumor cells recognition, and T-cell responses [1]. From the 1980s onward, members of type-I IFN family have been the standard care as immunotherapeutic agents in cancer therapy [2,3]. In particular, IFNα has been approved by the US Food and Drug Administration (FDA) for cancer. To date, pharmaceutical companies produce several types of recombinant and pegylated IFNα for clinical use; e.g., IFNα2a (Roferon-A, Roche), IFNα2b (Intron-A, Schering-Plough) and pegylated IFNα2b (Sylatron, Schering Corporation) [2] for treatment of hairy cell leukemia, melanoma, renal cell carcinoma, Kaposi's sarcoma, multiple myeloma, follicular and non-Hodgkin lymphoma, and chronic myelogenous leukemia [2,4,5]. Human IFNβ (Feron, Toray ltd.) has also been approved in Japan to treat glioblastoma, medulloblastoma, astrocytoma, and melanoma [5].

Despite the pleiotropic (e.g., antiangiogenic, immunomodulatory, differentiation-inducing, antiproliferative, and proapoptotic) actions of IFNα against malignancies [2,6]. It is still unclear why IFNα treatment is only effective in a subtype of patients (e.g.*,* melanoma), whilst might promote tumor progression in another subset [7]. Therefore, the administration of IFNα has been later surpassed by more effective and less toxic agents. For example, drugs such as thalidomide, lenalidomide and bortezomib are more recommended for myeloma treatment [2,6], while imatinib and tyrosine kinase inhibitors are more prevalently used for chronic myelogenous leukemia [2,6]. Additionally, vascular-endothelial growth factor (VEGF) and mammalian target of rapamycin (mTOR) inhibitors supersede for treatment of renal cell carcinoma. Nevertheless, no other treatment has shown superior efficacy to IFNα in the adjuvant phase of malignant melanoma yet [2]. Recently, the use of type-I IFNs has attracted attention once more in cancer therapy [8,9]. It has been shown that type-I IFNs have a synergistic effect on checkpoint blockade and adoptive T-cell immunotherapies by increasing the proliferation and cytotoxicity of T-cells, the maturation and cross-priming capacity of dendritic cells and stimulating NK cells to kill tumor cells [8,9]. In addition, advances in immunotherapy led to the emergence of cancer immunoediting which can serve as a framework to re-evaluate the IFNα's immunological role in tumor development and immunotherapy [1]. For example, in 2020, a phase I/II clinical trial showed that adoptive cell therapy with tumor-reactive T cells in combination with a mild IFNα regimen could increase the median overall survival in metastatic refractory melanoma patients from non-responders (7 months) to responders (36 months) [10].

In the human genome, a cluster of thirteen functional IFN genes is located at the 9p21.3 cytoband over approximately 400 kb including coding genes for IFNα (*IFNA1, IFNA2, IFNA4, IFNA5, IFNA6, IFNA7, IFNA8, IFNA10, IFNA13, IFNA14, IFNA16, IFNA17* and *IFNA21*), IFNω (*IFNW1*), IFNɛ (*IFNE*), IFNк (*IFNK*) and IFNβ (*IFNB1*), plus 11 IFN pseudogenes [2,11]. Among 19 cancer types, prevalent homozygous deletion of IFN gene cluster has been observed in high frequencies (7–31%) indicating that deletion of type-I IFN genes exacerbates overall or disease-free survival rates [11]. Defects in interferon signaling pathways have been suggested to induce mechanism of resistance to immunotherapy in prostate cancer cell lines [12] i.e., copy number deletion of IFN genes activates oncogenic pathways and repress immune signaling pathways by both promoting tumorigenesis and helping tumor cells to evade immunosurveillance [11]. Copy number deletion of the IFN  gene cluster may also leads to a worse prognosis in melanoma patients [13] and could be useful as a prognostic marker to predict resistance to immunotherapy (e.g., anti-CTLA4 treatment) [11]. Consequently, it is suggested that individuals with deletions may benefit from combinations of IFNα with T cell-directed therapies [13].

In this study, we investigate the association of copy number alteration (CNA) of the IFN gene cluster with the mortality and survival of patients with different cancer types. This individual patient data meta-analysis aims to predict which CNA subtype in different cancers could benefit from interferon α/β therapy and fills a gap in our understanding of type I interferon gene copy number on cancer progression and treatment.

## Methods

The individual patient data meta-analysis is based on data obtained from the cBioportal database (www.cbioportal.org) containing published and unpublished data mostly from The Cancer Genome Atlas (TCGA) [14,15]. Only individuals without a prior history of cancer, complete information on the CNA of the IFN gene cluster, and 5-y follow-up were included in this study.

### Data extraction

Datasets of demographics, clinical information related to cancer (including anatomical location and histological subtype), and cancer genomics have been extracted for all individuals (www.cbioportal.org / faq.jsp) [14,15]. Data obtained from cBioportal for the CNA categories were computed using Genomic Identification of Significant Targets in Cancer (GISTIC) version 2.0 [16]; this method has been described in more detail previously [17].

### Data analysis

A meta-analysis of individual patient data was conducted in Stata/MP14.2 (StataCorp, USA). Two meta-analytical approaches were used to determine the stability and consistency of the results. The main outcome was 5-y mortality for each anatomical location of cancer, expressed as odd ratios (OR) and 95 % confidence intervals (CI), using diploidy (normal) as the reference group [18]. Descriptive statistics are shown as the number of individuals and proportions (%). In order to maintain sufficient statistical power, shallow (-1) and deep (-2) deletion was combined for the meta-analyses, as well as gain (+1) and amplification ≥+2); respectively referred to as “deletion” and “amplification.” ORs above 1 imply a higher risk of mortality in the deletion/amplification group, compared to the reference; whilst lower values propose a protective effect. If a 95% CI includes the value of 1 (indicating no difference), differences with the reference group are statistically insignificant.

The first meta-analytic approach was based on random effect modeling using the ipdmetan package in Stata. This two-stage individual patient data meta-analysis pools and visualizes the effect of CNA on the risk of death (yes or no) within 5 y after diagnosis, weighted for the different anatomical locations, and the results are presented as forest plots [19]. I-squared statistics were used to quantify statistical heterogeneity, with values <50%, 50% to 75%, and >75% defined as low, moderate, and high heterogeneity, respectively [20]. This approach does not allow for adjustment for confounding or interaction. Therefore, a second meta-analytic method was implemented, multivariable logistic regression analyses (one-step approach) [18]. For each anatomical location, three models were used. Model 1 was crude (unadjusted); Model 2 was adjusted for sex, age, and calendar period; and Model 3 was additionally adjusted for interaction with tumor stage. Analyses were only presented if at least 10 individuals were included in each risk group, and subgroups with zero deaths were omitted. All analyses are based on complete-case analyses.

### Survival analysis

Information regarding the association of CNA of IFN gene cluster and patients’ survival time in cancer was analyzed using TCGA PanCancer Atlas Studies database, available at cBioportal. Only patients with the CNA of the IFN gene cluster and overall survival data (*n* = 10,712) in 32 cancer type were analyzed to observe the prognostic value using Kaplan–Meier Plots. All reported *P <* 0.05 were considered statistically significant.

## Results

### Description of CNA in different cancer types

This study includes 9937 patients, for whom information on CNA outcome was available (Table 1). Of these, 55% were female, approximately half were older than 60 y and 26% were diagnosed between 2011 and 2013 (Table 1). Tumors were stage 0–I (in-situ) (18%), stage II (10%), stage III (15%), stage IV (6%), and information on stage was missing for 50%. In total, 24 different anatomical locations were represented, with breast (15%), brain (9%), lung (8%), kidney (7%), and prostate (7%) cancer as the largest groups.

**Table 1:** Clinical and tumor characteristics and 5-y prognosis of the entire cohort, by copy number alteration (CNA) category.

	Deletion (-1/-2)		Diploid (Reference)		Amplification (+1/+2)		Total	
	No.	%	No.	%	No.	%	No.	%
**Total**	5915	59.5	3122	31.4	900	9.05	9937	100.0
**Sex**								
Female	1552	49.7	3380	57.1	522	58.0	5454	54.9
Male	1563	50.1	2511	42.5	377	41.9	4451	44.8
Missing	7	0.2	24	0.4	1	0.1	32	0.3
**Age (y)**								
<40	235	7.5	689	11.6	73	8.1	997	10.0
40–49	321	10.3	844	14.3	112	12.4	1277	12.9
50–59	728	23.3	1353	22.9	187	20.8	2268	22.8
60–69	886	28.4	1521	25.7	242	26.9	2649	26.7
70–95	842	27.0	1282	21.7	236	26.2	2360	23.7
Missing	110	3.5	226	3.8	50	5.6	386	3.9
**Calendar period**								
1978–2005	786	25.2	841	14.2	205	22.8	1832	18.4
2006–2008	524	16.8	858	14.5	142	15.8	1524	15.3
2009–2010	496	15.9	956	16.2	158	17.6	1610	16.2
2011–2013	807	25.8	1689	28.6	237	26.3	2733	27.5
Missing	509	16.3	1571	26.6	158	17.6	2238	22.5
**Tumor stage**								
Stage 0–I	528	16.9	1138	19.2	112	12.4	1778	17.9
Stage II	444	14.2	457	7.7	87	9.7	988	9.9
Stage III	629	20.1	709	12.0	181	20.1	1519	15.3
Stage IV	271	8.7	273	4.6	92	10.2	636	6.4
Missing	1250	40.0	3338	56.4	428	47.6	5016	50.5
**Anatomical location**								
Adrenal glands	8	0.3	102	1.7	4	0.4	114	1.1
Bladder	40	1.3	25	0.4	16	1.8	81	0.8
Blood	6	0.2	223	3.8	8	0.9	237	2.4
Brain	443	14.2	464	7.8	32	3.6	939	9.4
Breast	327	10.5	1100	18.6	82	9.1	1509	15.2
Cervical	55	1.8	180	3.0	51	5.7	286	2.9
Colorectal	70	2.2	353	6.0	109	12.1	532	5.4
Esophagus	102	3.3	50	0.8	16	1.8	168	1.7
Eyes	8	0.3	62	1.0	5	0.6	75	0.8
Head and Neck	205	6.6	176	3.0	91	10.1	472	4.7
Kidney	168	5.4	550	9.3	21	2.3	739	7.4
Liver	113	3.6	193	3.3	18	2.0	324	3.3
Lung	521	16.7	217	3.7	79	8.8	817	8.2
Mesenchyme	70	2.2	94	1.6	31	3.4	195	2.0
Mesothelium	33	1.1	31	0.5	1	0.1	65	0.7
Ovarian	254	8.1	178	3.0	124	13.8	556	5.6
Pancreas	74	2.4	61	1.0	4	0.4	139	1.4
Prostate	73	2.3	551	9.3	75	8.3	699	7.0
Skin	229	7.3	88	1.5	13	1.4	330	3.3
Stomach	145	4.6	225	3.8	44	4.9	414	4.2
Testicular	52	1.7	61	1.0	17	1.9	130	1.3
Thymus	6	0.2	97	1.6	9	1.0	112	1.1
Thyroid	20	0.6	443	7.5	4	0.4	467	4.7
Uterus	100	3.2	391	6.6	46	5.1	537	5.4
**Five-y outcome**								
Alive	1883	60.3	4704	79.5	622	69.1	7209	72.5
Died	1239	39.7	1211	20.5	278	30.9	2728	27.5
**Five-y recurrence**								
Disease free	1150	36.8	3000	50.7	407	45.2	4557	45.9
Recurred/Progressed	391	12.5	564	9.5	101	11.2	1056	10.6
Died	1239	39.7	1211	20.5	278	30.9	2728	27.5
Unclear	342	11.0	1140	19.3	114	12.7	1596	16.1

**Fig. 1:**
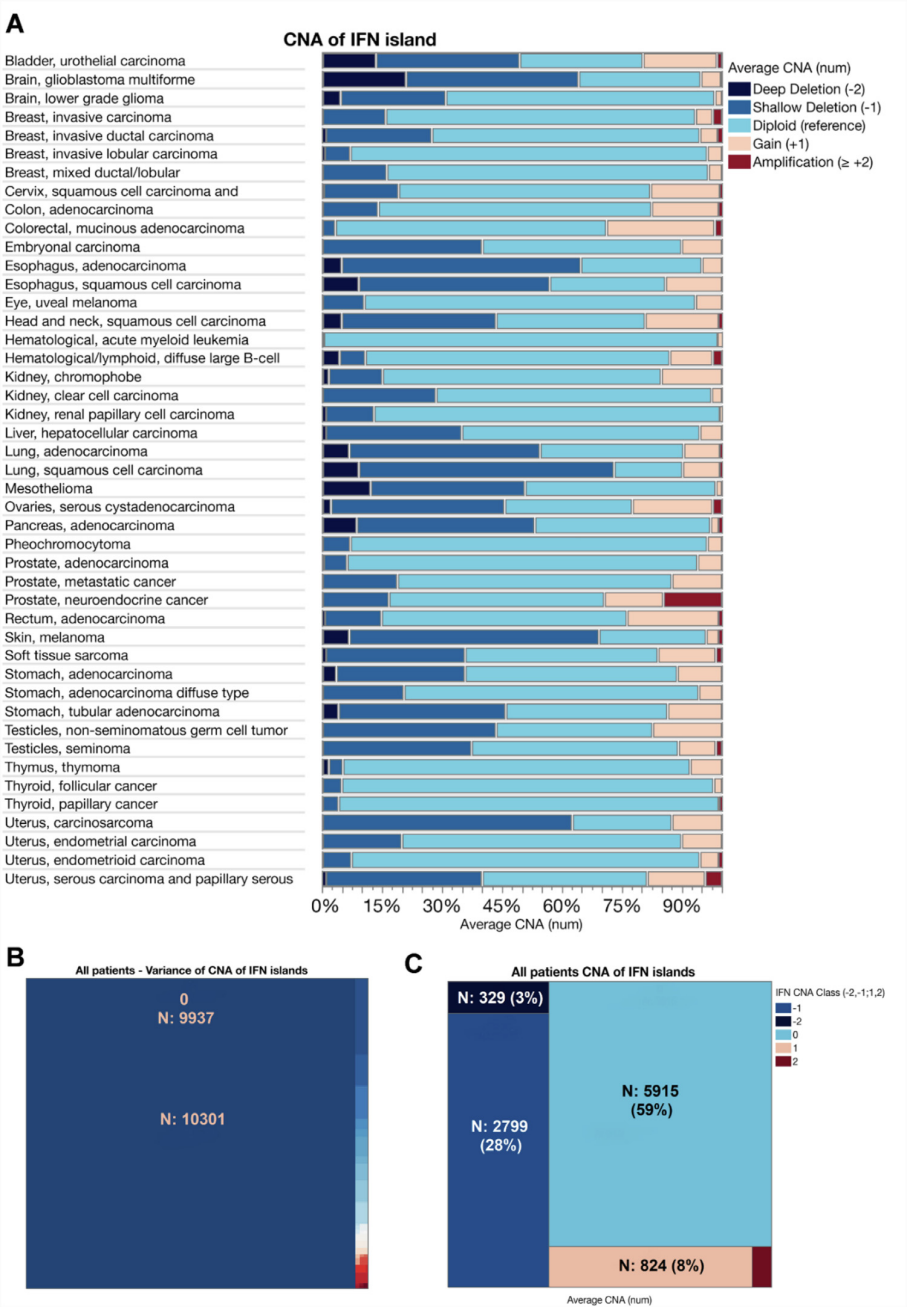
Copy number alteration of IFN gene cluster among cancer types. (A) Distribution of IFN gene cluster CNA per cancer type. (B) The variance of IFN gene cluster CNA. (C) IFN CNA classes (-2: deep deletion, -1: shallow deletion, 0: diploid, +1: gain, ≥ +2: amplification). Numbers in brackets indicate the number of patient samples per cancer type.

Analysis of the 9p21.3 IFN gene cluster showed that shallow (-1) and deep (-2) deletions represented a large proportion of tumors whereas gain and amplification were less frequent (Fig. 1A). Analysis of the CNA variance for genes contained within the IFN gene cluster showed that 9937 of the 10,301 patient samples were homogeneous (96%) (Fig. 1B). Average CNA of IFN gene cluster in all patients shows the percentage of diploidy (59%), gains (8%) amplification (1%), deep deletion (3%), and shallow deletion (28%), (Fig. 1C). In a total of 9,937 patients; diploidy, gain/amplification and deletions of IFN gene cluster represented respectively 60%, 9% and 31% of the population (Table 1). Respectively 57%, 58% and 50% of the patients with diploidy, amplification and deletion of the IFN gene cluster were female (Table 1). The highest proportion of gain/amplification was seen among ovarian (22%), colorectal (20%) and bladder (20%) cancers. Whereas the highest proportion of deletion was seen among skin (69%), lung (63%), esophagus (61%), pancreas, (53%) and mesothelium (51%) cancers (Table 2).

**Table 2:** Distribution of deletion, diploidy and amplification by anatomical location, indicating which proportion of each location has normal or abnormal copy number alterations (CNA).

Anatomical location	Deletion (-1/-2)		Diploid (reference)		Amplification (+1/ > +2)	
	No.	%	No.	%	No.	%
Adrenal glands	8	7.0	102	89.5	4	3.5
Bladder	40	49.4	25	30.9	16	19.8
Blood	6	2.5	223	94.1	8	3.4
Brain	443	47.2	464	49.4	32	3.4
Breast	327	21.7	1,100	72.9	82	5.4
Cervical	55	19.2	180	62.9	51	17.8
Colorectal	70	13.2	353	66.4	109	20.5
Esophagus	102	60.7	50	29.8	16	9.5
Eyes	8	10.7	62	82.7	5	6.7
Head and Neck	205	43.4	176	37.3	91	19.3
Kidney	168	22.7	550	74.4	21	2.8
Liver	113	34.9	193	59.6	18	5.6
Lung	521	63.8	217	26.6	79	9.7
Mesenchyme	70	35.9	94	48.2	31	15.9
Mesothelium	33	50.8	31	47.7	1	1.5
Ovarian	254	45.7	178	32.0	124	22.3
Pancreas	74	53.2	61	43.9	4	2.9
Prostate	73	10.4	551	78.8	75	10.7
Skin	229	69.4	88	26.7	13	3.9
Stomach	145	35.0	225	54.3	44	10.6
Testicles	52	40.0	61	46.9	17	13.1
Thymus	6	5.4	97	86.6	9	8.0
Thyroid	20	4.3	443	94.9	4	0.9
Uterus	100	18.6	391	72.8	46	8.6
**Total**	**3,122**	**31.4**	**5,915**	**59.5**	**900**	**9.1**

Age distribution was similar in all groups, yet individuals with advanced tumor stage (III–IV) were overrepresented in the group with copy number deletion (20% and 8%) and amplification (20% and 10%) compared to patients carrying diploidy (12% and 5%).

In 6 of the 24 anatomical locations, the large majority of the cancers presented either deletion or amplifications, with diploidy only present in 37% (head and neck cancer), 31% (bladder), 32% (ovaries), 30% (esophagus), 27% (skin), and 27% (lung).

### Clinical characteristics

All patients having heterogeneous CNAs within the IFN gene cluster and those with a prior malignancy or incomplete 5-y follow-up information were excluded. In total, 24 different anatomical locations were reported with breast (15%), and brain tumors (9%) being most common.

In total, 3122 individuals (31%) were diploid for the IFN gene cluster (Table 1), 900 patients (9%) showed gain or amplification and 5915 individuals (59%) had deletions. Women presented more frequently with diploidy and amplification (57% and 58%) than men (42% and 41%) (*P* < 0.0001), and the proportion of diploidy increased by age (12% in < 40 y, 22% in ≥ 70 y; *P* < 0.0001). Diploidy was more common in breast cancers (19%) followed by kidney and prostate cancers (9%). The IFN gene cluster deletion was especially common in the lung (17%) and brain (14%). Diploidy was more common in stage 0–I (19%) compared to stage IV (5%) (*P* < 0.0001) (Table 1).

### Prognosis per anatomical location

**Fig. 2:**
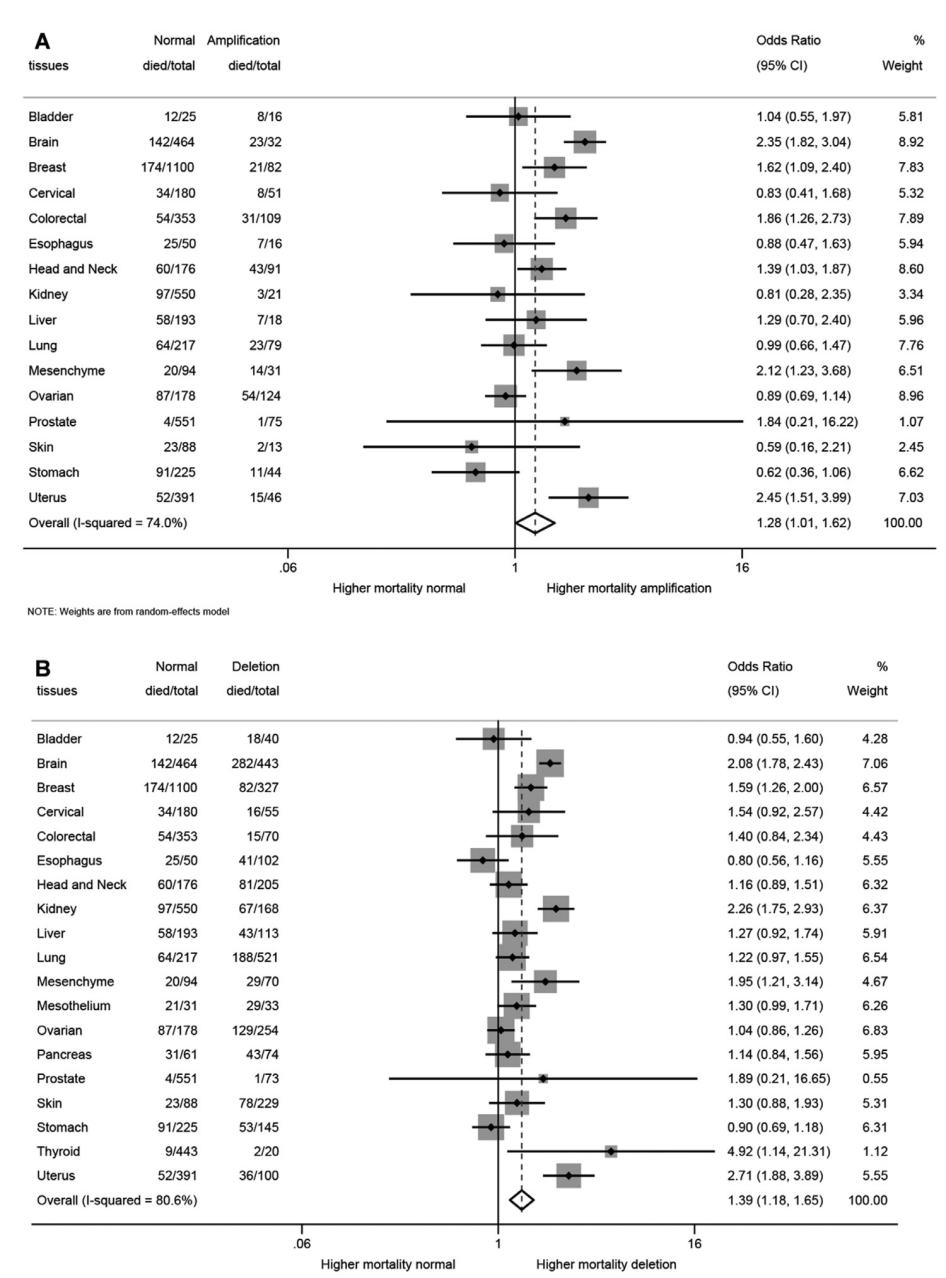
Forest plots assessing the association between IFN gene cluster. (A) Amplification and (B) Deletion. 5-y mortality per cancer type using diploidy as a reference. The numbers in the columns refer to the total number of individuals presenting with each cancer type, and the number who died within 5 y, for the total group of individuals, those with diploidy (reference) and those with amplification (A) or deletion (B). The diamond represents the average of studies. Weights are derived from a random-effects model. CI, confidence interval; OR, odds ratio.

The forest plots for amplification and deletion per anatomical location are presented in Fig. 2. This two-step meta-analysis approach shows that, compared to diploidy as a reference, amplification was associated with a significantly increased mortality for 6 cancer types, in particular for the uterus (OR = 2.45), brain (OR = 2.35), and mesenchyme (OR = 2.12), colorectal (OR = 1.86), breast (OR = 1.62), and head and neck (OR = 1.39) cancers (Fig. 2A). For deletions, loss of the IFN gene cluster was associated with a significantly increased mortality in 9 cancer types, including thyroid (OR = 4.92), uterus (OR = 2.71), kidney (OR = 2.26), brain (OR = 2.08), mesenchyme (OR = 1.95), breast (OR = 1.59), mesothelium (OR = 1.30), liver (OR = 1.27), and lung (OR = 1.22) (Fig. 2B).

The one-step approach provided similar results, and all three models were presented for each anatomical location if feasible (Table 3). For deletion, model-2 showed a significantly increased 5-y mortality for cancers of the uterus (OR = 3.35), kidney (OR = 2.82), brain (OR = 2.46), and liver (OR = 1.72). After full adjustment (model 3, in half of the cohort with complete tumor stage), none of the results was confirmed; however, deletion remained significantly associated with a worse prognosis in the total cohort (OR = 2.41) (Table 3).

**Table 3:** The 5-y mortality associated with copy number alterations (CNA) per anatomical location, calculated by multivariable logistic regression and presented as number (%) and odds ratio's (OR) and 95% confidence intervals (CI).

	Deletion (-1/-2)				Diploid (reference)				Amplification (+1/+2)				Model 1*				Model 2⁎⁎				Model 3⁎⁎⁎			
Cancer Type	Alive		Died		Alive		Died		Alive		Died		All Deletions		Gain or Amplification		All Deletions		Gain or Amplification		All Deletions		Gain or Amplification	
	No.	%	No.	%	No.	%	No.	%	No.	%	No.	%	OR	95% CI	OR	95% CI	OR	95% CI	OR	95% CI	OR	95% CI	OR	95% CI
Adrenal	7	88	1	12.5	101	99	1	1	4	100	0	0	–	–	–	–	–	–	–	–	–	–	–	–
Bladder ×× Blood	22	55	18	45	13	52	12	48	8	50	8	50	0.9	[0.33,2.41]	1.1	[0.31,3.80]	0.4	[0.11,1.40]	1.1	[0.26,5.01]	0.2	[0.02,1.97]	0	[0.00,0.99]
	5	83	1	16.7	93	42	130	58	6	75	2	25	–	–	–	–	–	–	–	–	–	–	–	–
Brain	161	36	282	63.7	322	69	142	31	9	28	23	72	4	[3.01,5.24]	5.8	[2.62,12.84]	2.5	[1.71,3.54]	4.1	[1.46,11.65]	–	–	–	–
Breast	245	75	82	25.1	926	84	174	16	61	74	21	26	1.8	[1.32,2.40]	1.8	[1.09,3.09]	–	–	–	–	–	–	–	–
Cervical	39	71	16	29.1	146	81	34	19	43	84	8	16	1.8	[0.88,3.52]	0.8	[0.34,1.85]	1.5	[0.73,3.23]	0.7	[0.28,1.62]	–	–	–	–
Colorectal	55	79	15	21.4	299	85	54	15	78	72	31	28	1.5	[0.80,2.86]	2.2	[1.33,3.65]	1.9	[0.94,3.67]	2.5	[1.47,4.32]	–	–	–	–
Esophagus	61	60	41	40.2	25	50	25	50	9	56	7	44	0.7	[0.34,1.33]	0.8	[0.25,2.41]	–	–	–	–	–	–	–	–
Eyes	4	50	4	50	47	76	15	24	4	80	1	20	–	–	–	–	–	–	–	–	–	–	–	–
Head-Neck	124	61	81	39.5	116	66	60	34	48	53	43	47	1.3	[0.83,1.92]	1.7	[1.03,2.90]	1.3	[0.83,2.06]	1.8	[1.04,3.17]	5.6	[0.33,93.17]	–	–
Kidney	101	60	67	39.9	453	82	97	18	18	86	3	14	3.1	[2.12,4.52]	0.8	[0.22,2.69]	2.8	[1.87,4.25]	0.5	[0.14,1.77]	1.4	[0.51,3.84]	–	–
Liver	70	62	43	38.1	135	70	58	30	11	61	7	39	1.4	[0.88,2.33]	1.5	[0.55,4.01]	1.7	[1.00,2.94]	1.7	[0.58,4.80]	1.5	[0.66,3.62]	0.7	[0.06,7.84]
Lung	333	64	188	36.1	153	71	64	30	56	71	23	29	1.4	[0.96,1.90]	1	[0.56,1.73]	1.3	[0.91,1.88]	1	[0.56,1.86]	1.3	[0.77,2.29]	0.8	[0.30,1.94]
Mesenchyme	41	59	29	41.4	74	79	20	21	17	55	14	45	2.6	[1.32,5.20]	3.1	[1.29,7.22]	1.9	[0.91,4.13]	1.8	[0.70,4.71]	–	–	–	–
Mesothelium	4	12	29	87.9	10	32	21	68	1	100	0	0	3.5	[0.95,12.52]	–	–	–	–	–	–	–	–	–	–
Ovarian	125	49	129	50.8	91	51	87	49	70	57	54	44	1.1	[0.74,1.58]	0.8	[0.51,1.28]	1	[0.63,1.49]	0.8	[0.45,1.25]	2.1	[0.12,35.02]	–	–
Pancreas	31	42	43	58.1	30	49	31	51	1	25	3	75	1.3	[0.68,2.66]	2.9	[0.29,29.49]	–	–	–	–	–	–	–	–
Prostate	72	99	1	1.4	547	99	4	1	74	99	1	1.3	1.9	[0.21,17.23]	1.9	[0.20,16.76]	4.2	[0.44,40.55]	4.3	[0.43,42.63]	–	–	–	–
Skin	151	66	78	34.1	65	74	23	26	11	85	2	15	1.5	[0.84,2.53]	0.5	[0.11,2.49]	1.8	[0.98,3.14]	0.6	[0.11,2.87]	3.6	[0.71,17.90]	–	–
Stomach	92	63	53	36.6	134	60	91	40	33	75	11	25	0.9	[0.55,1.30]	0.5	[0.24,1.02]	1	[0.62,1.55]	0.6	[0.27,1.31]	0.4	[0.07,2.48]	–	–
Testicular	52	100	0	0	59	97	2	3	17	100	0	0	–	–	–	–	–	–	–	–	–	–	–	–
Thymus	6	100	0	0	92	95	5	5	8	89	1	11	–	–	–	–	–	–	–	–	–	–	–	–
Thyroid	18	90	2	10	434	98	9	2	4	100	0	0	5.4	[1.08,26.62]	–	–	–	–	–	–	–	–	–	–
Uterus	64	64	36	36	339	87	52	13	31	67	15	33	3.7	[2.22,6.06]	3.2	[1.59,6.24]	3.4	[1.97,5.68]	3.1	[1.52,6.39]	2.5	[0.98,6.12]	1.7	[0.45,6.66]
Total	1883	60	1239	39.7	4704	80	1211	21	622	69	278	31	2.6	[2.32,2.81]	1.7	[1.49,2.03]	2.5	[2.27,2.84]	1.7	[1.41,2.02]	2.4	[1.79,3.20]	1	[0.51,1.86]
Total §													2.6	[1.74,3.76]	1.7	[1.24,2.43]	2.5	[1.70,3.78]	1.7	[1.22,2.37]	2.4	[1.66,3.51]	1	[0.54,1.89]

⁎ Model 1 (unadjusted).

⁎⁎ Model 2 (adjusted for age. sex, calendar period).

⁎⁎⁎ Model 3 (adjusted for age, sex, calendar period and Interaction with tumor stage. § adjusted for clustering by tissue. Note: if less than 10 individuals in the deletion or amplification group, results are not presented.

For amplification, model-2 found significant associations for cancers of the brain (OR = 4.12), uterus (OR =3.11), colorectal (OR = 2.52), and head and neck (OR = 1.82) cancers; again, not confirmed in model 3 (Table 3).

### Overall prognosis

At 5 y after diagnosis, 27% of patients have died, 46% were disease-free, and 11% had a recurrence but were still alive. Recurrence information was missing in 11% of individuals who survived. Of those who died, 20% presented with IFN gene cluster diploidy. Of those who were alive, 51% presented diploidy (*P* < 0.00001) (Table 1).

The one-step meta-analysis approach was used to assess if the effects of IFN gene cluster ploidy on mortality remained after adjustment for confounding and interaction using diploidy as a reference. The unadjusted 5-y mortality (model 1, *n* =  9937) showed similar results as above, with respectively 256% (OR = 2.56, 95% CI 1.74–3.76) and 174% (OR = 1.74, 95% CI 1.24–2.43) increased risks of death for deletion and amplification compared to the diploid group (Table 3). After adjustments for age, sex, calendar period, and clustering by study (model 2, *n* = 7666), the results remained stable yet lost significance. Since the interaction between IFN cluster ploidy and tumor stage was present (*P* = 0.0031). Model 3 (*n* = 4859) is additionally adjusted for interaction with tumor stage, resulting in doubled risks among those with deletion (OR = 2.41, 95% CI 1.66–3.51), yet no difference for those with amplification (OR = 1.01, 95% CI 0.54–1.89) (Table 3).

### Survival

**Fig. 3:**
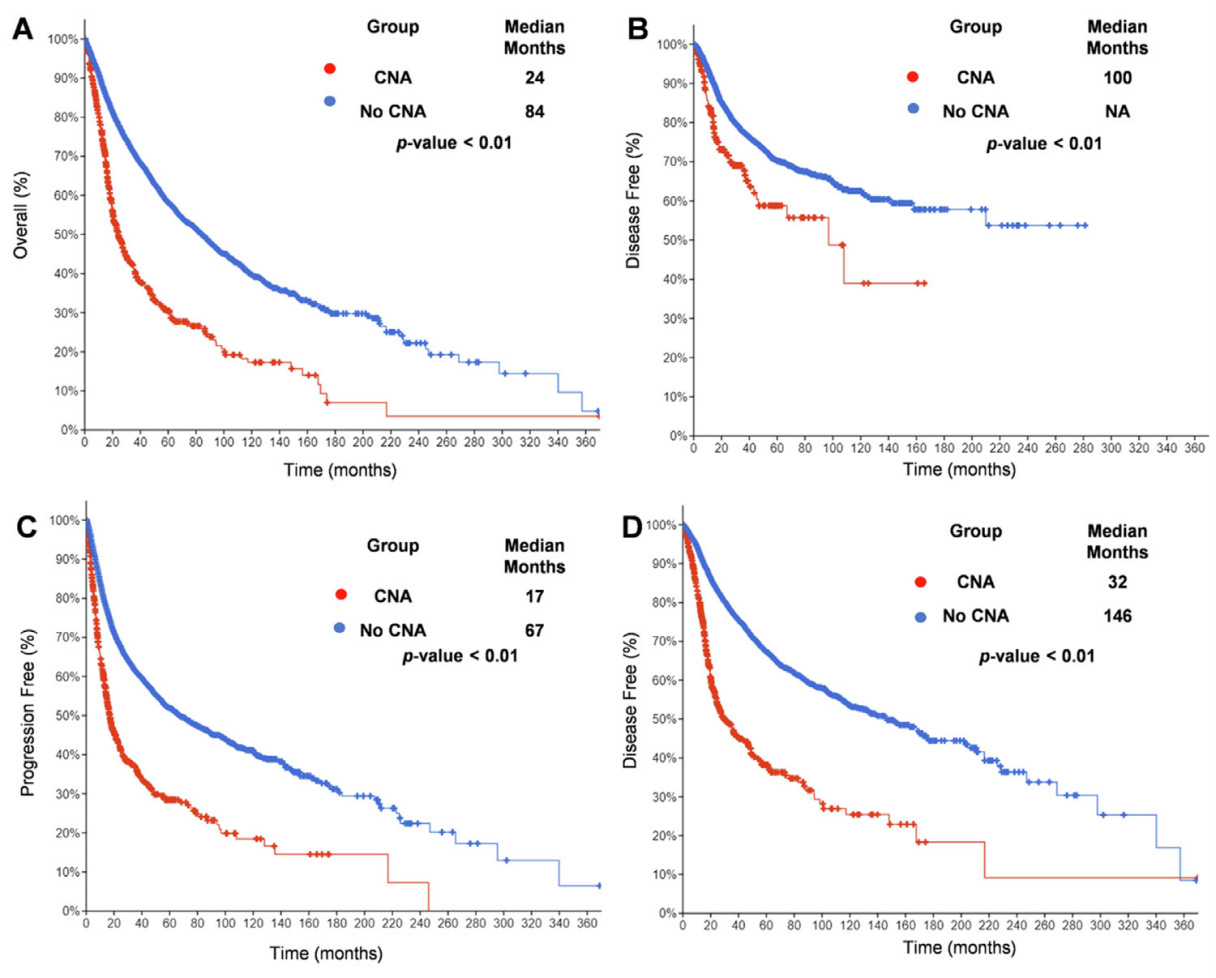
Overall (A), disease-free (B), progression-free (C) and disease-specific (D) survival of 35 cancer types extracted from cBioportal, TCGA combined PanCancer Atlas. CAN, copy number alteration (of IFN gene cluster); NA, not available. Note: if less than 10 individuals in the cohort, results are not presented.

Screening of available data of 10,712 patients from TCGA combined PanCancer Atlas using cBioportal shows 7% CNA of IFN gene cluster in a total of 32 cancer types included in the study. In addition, overall, disease-free, progression-free, and disease-specific survival in groups with CNA of IFN gene cluster are significantly decreased to 24, 100, 17, and 32 months (median), respectively (Fig. 3).

**Fig. 4:**
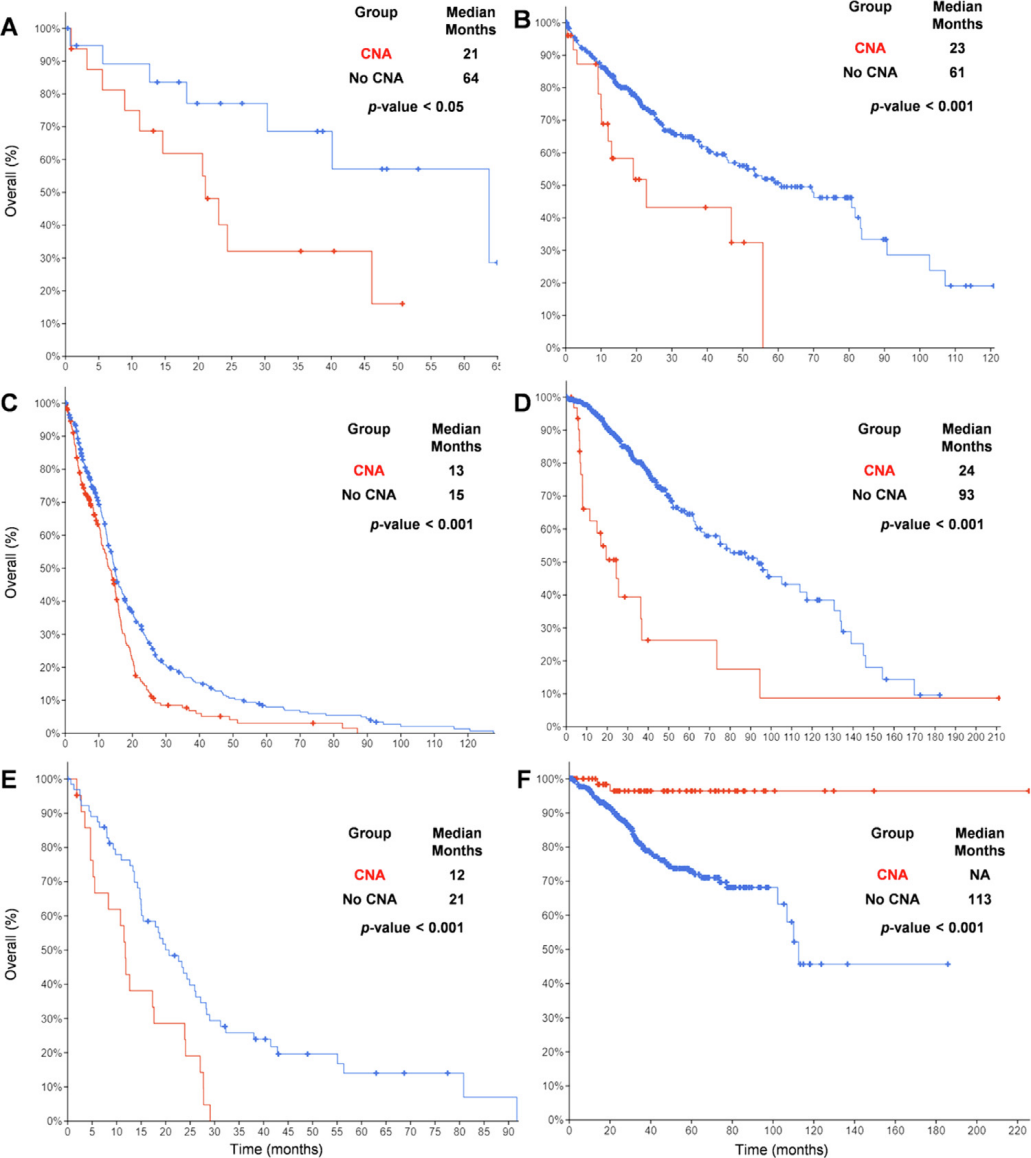
Overall survival of 6 cancer types shows a statistically significant difference between patients with CNA and no-CNA of IFN gene cluster. (A) Cholangiocarcinoma, (B) liver hepatocellular carcinoma, (C) glioblastoma multiforme, (D) brain low-grade glioma, (E) mesothelioma, (F) Uterine corpus endometrial carcinoma. CNA, copy number alteration (of IFN gene cluster); NA, not available.

Furthermore, overall survival is significantly altered in 6 out of 35 cancer types due to the CNA of the IFN gene cluster in patients. The overall survival is decreased in cholangiocarcinoma (Fig. 4A), liver hepatocellular carcinoma (Fig. 4B), glioblastoma multiforme (Fig. 4C), brain low-grade glioma (Fig. 4D) and mesothelioma (Fig. 4E). In contrast, the overall survival is increased in uterine corpus endometrial carcinoma (Fig. 4F).

## Discussion

This study shows consistent results using two statistical approaches, and a Pan-Cancer analysis. The CNA of the IFN gene cluster (both in the deletion and amplification forms) was associated with increased mortality of cancer patients. In addition, deletion of the IFN gene cluster was more prevalent than diploidy and amplification. Patients with deletion of the IFN gene cluster show higher mortality in 9 out of 24 cancer types including the brain, breast, cervical, kidney, liver, lung, mesenchyme, mesothelioma, thyroid, and uterus. While patients with amplification of the IFN gene cluster show higher mortality in 6 cancer types including brain, breast, colorectal, head and neck, mesenchyme, and uterus. No association was found for the other cancer types, but power may have been too limited. In a total of 35 cancer types provided by TCGA/cBioportal, survival (overall, disease-free, progression-free, and disease-specific) in patients with CNA of IFN gene cluster are less in comparison to unaltered individuals. In particular, the overall survival for cholangiocarcinoma, liver hepatocellular carcinoma, glioblastoma multiforme, brain low-grade glioma, and mesothelioma is less, yet higher mortality was found for liver, brain, and lung cancers.

This large individual patient data meta-analysis is based on the cBioportal and TCGA with high quality/valid data. The cBioportal is an open-access resource for interactive exploration of cancer omics data-sets including TCGA, empowering researchers to translate these valuable data-sets into biological insights and clinical applications [14]. However, our study may have limited power for the different histological subtypes, and possible selection bias since some cancer types may be over-represented in the cohort compared to cancer distribution in the total population. We could adjust for age, sex, calendar period and to a certain extent for tumor stage (despite a large amount of missing value) but other “residual” confounders may play a role as well i.e., these results indicate associations, but not causations.

Nevertheless, deletion of the IFN gene cluster might play a more deleterious role in patients due to the lower level of type-I interferon expression leading to less immunosurveillance in the tumor microenvironment. Because type-I IFNs exerts their anti-tumor activity through driving the high maturation status of dendritic cells, impacting cytotoxic T lymphocytes and NK cell activation, inducing tumor cell death and inhibiting angiogenesis [21], i.e., lower expression of type-I IFNs can impede immunosurveillance.

Among the cancer types which IFN α/β therapy has been approved for, only mortality of kidney and brain cancer types was associated with CNA of IFN gene cluster. In contrast, the mortality of hematological malignancies (e.g.*,* multiple myeloma, follicular and non-Hodgkin lymphoma, and chronic myelogenous leukemia) was not associated with CNA of IFN gene cluster and skin cancer had limited power. It has already been shown that subset of melanoma patients showing deletion of IFN gene cluster is resistant to anti-CTLA4 immunotherapy proposing that deletion of IFN gene cluster can be used a prognostic biomarker for immunotherapy resistance [11]. In this regard, phase II clinical trial of combinational therapy of anti-CTLA4 (Ipilimumab) with recombinant IFNα in treating melanoma patients is currently undergoing (ClinicalTrials.gov Identifier: NCT01708941) [22] suggesting that such a regimen might have potential benefit in cancer types with CNA of IFN gene cluster (e.g., kidney and brain cancers). For example, two phase II clinical trials of IFNα with temozolomide in patients with recurrent glioblastoma multiforme demonstrated improvement in six months progression-free survival outcomes [23].

In 2018, anti-CTLA4 (ipilimumab) plus nivolumab was approved by the FDA for renal cell carcinoma treatment [24]. Our results also show that a subset of patients with deletion of IFN gene cluster (23%) in kidney cancer has a significantly higher mortality. Thus, deletion of the IFN gene cluster might be potentially used as a prognostic biomarker for anti-CTLA4 immunotherapy resistance in renal cell carcinoma as well. Furthermore, in the phase II clinical trial, a combination of IFNα and chemotherapy (Oxaliplatin/Adriamycin/5-Fluorouracil) showed manageable toxicity and improved survival of patients with advanced hepatocellular carcinoma [25]. Therefore, deletion of the IFN gene cluster in liver cancer might also be used as a potential prognostic biomarker for IFNα therapy in liver cancer.

In general, the new strategies for application of interferons are included to the in-vivo use of  IFNα as immune adjuvants of cancer vaccines, and the combination of certain chemotherapies with IFNα adjuvanted cancer vaccines [26]. Personalized medicine/ immunotherapy is another emerging area for application of interferons, fostering the development of specialized treatments for each subtype of cancer, based on the measurement and exploitation of patients’ omics data (e.g., genomics, transcriptomics, metabolomics, proteomics) [27,28]. In this light, understanding the alteration of IFN gene cluster helps to predict the resistance to cancer therapy outcome as a prognostic biomarker in companion diagnostics. Particularly, for cancer types such as brain, kidney, skin, and hematologic malignancies which IFNα/β therapy is already in clinical use. Furthermore, the information about alteration of IFN gene helps to expand the clinical application of interferon α/β therapy in the type of cancers showing higher mortality associated with CNA of the IFN gene cluster e.g., breast, and uterine cancers.

## Conclusions

This large individual patient data meta-analysis indicates that CNA of the IFN gene cluster is prevalent in cancer. Two statistical approaches showed that notably amplification and deletion of the IFN gene cluster are significantly associated with increased mortality in at least 6 and 9 cancer types, respectively. PanCancer TCGA analysis using cBioportal also showed CNA of IFN gene cluster is significantly associated with decreased survival in liver, brain, and mesothelioma cancers. Therefore, CNA of the IFN gene cluster can be suggested as a useful biomarker to predict the prognosis of patients (e.g.*,* liver and renal cancers) and also as a potential companion diagnostic test to prescribe IFN α/β therapy and predict the outcome of immunotherapy for clinical use.

## Author's contributions

A.R. N.B, and M.DD. performed the experiments and analyses. A.R., N.B., M.B., and M.DD supervised the project. A.R., N.B., M.DD. wrote the manuscript. All authors of this work had full access to this study and the data and approved to submit the final manuscript.

## Declaration of Competing Interest

The authors declare that they have no competing interests.
